# Indirect estimates of cellular hydration and relative water content and their associations with muscle strength and physical function in older adults: a path analysis from the Pro-Eva study

**DOI:** 10.1186/s11556-025-00400-y

**Published:** 2026-02-20

**Authors:** Weslley Barbosa Sales, Giane Amorim Ribeiro-Samora, Paulo Eduardo e Silva Barbosa, Edgar Ramos Vieira, Gérson Fonseca Souza, Álvaro Campos Cavalcanti Maciel

**Affiliations:** 1 Lagoa Nova, Natal - RN, Brazil, 59078-970, Federal University of Rio Grande do Norte, Natal, Brazil; 2 Universidade Federal de Minas Gerais, Belo Horizonte, Brazil; 3 State University of Paraíba, Campina Grande, Brazil; 4 Florida International University, Miami, United States

**Keywords:** Hydration Status, Sarcopenia, Aging, Physical Performance

## Abstract

**Aim:**

This study aimed to examine the associations between clinical characteristics, body composition parameters, and functional outcomes in older adults, with a specific focus on bioelectrical impedance analysis (BIA)-derived estimates related to hydration status—intracellular water (ICW) and the total body water-to-weight ratio (TBW/BW%). Outcomes included handgrip strength (HGS), performance in the Short Physical Performance Battery (SPPB), and calf circumference (CC), used here as an indirect indicator of muscle mass and lower-limb function. ICW and TBW/BW% were selected instead of extracellular water (ECW) or the ICW/ECW ratio to minimize collinearity and emphasize hydration-related estimates with physiological relevance to muscle function in older adults.

**Methods:**

A cross-sectional study was conducted with 1,009 community-dwelling older adults (≥ 60 years) from Parnamirim, RN, Brazil. Clinical variables (sex, diabetes, hypertension, polypharmacy, and education ≥ 4 years), anthropometry (BMI), and hydration-related parameters estimated via single-frequency BIA (Biodynamics, model 450) were assessed. Structural equation modeling with path analysis estimated the standardized direct associations (β) of these variables with HGS, CC, and SPPB scores.

**Results:**

For HGS, female sex, ICW, and TBW/BW% were significant correlates (β = –6.66, β = 0.79, and β = 0.03, respectively). In the CC model, ICW, BMI, and diabetes were significant predictors (β = 0.26, β = 0.37, and β = –0.52, respectively). For SPPB, higher ICW, TBW/BW%, and education (≥ 4 years) were positively associated (β = 0.14, β = 0.07, and β = 0.44, respectively), while hypertension (β = –0.29) and polypharmacy (β = –0.69) showed negative associations. Model explained variances (R^2^) were 51.0% (HGS), 42.1% (CC), and 14.2% (SPPB).

**Conclusion:**

ICW, as an indirect hydration-related estimate derived from BIA, was consistently associated with muscle strength and functional measures. TBW/BW% demonstrated smaller but significant associations with global physical performance. These findings indicate that hydration-related parameters obtained from BIA may contribute to identifying functional vulnerability in older adults, although they should not be interpreted as direct markers of cellular integrity or muscle tissue properties.

## Introduction

The ability to maintain good functional health in older adults depends on several factors, among which preserving skeletal muscle mass and adequate cellular hydration are fundamental [1–3]. With advancing age, substantial changes in body composition occur, including reductions in muscle mass, increases in adiposity, and notable declines in intracellular water (ICW). ICW is a biologically meaningful component of fat-free mass and is related to cellular hydration and the metabolic activity of muscle tissue [4, 5].

Although ICW has often been described as an indicator of cellular hydration, in the context of bioelectrical impedance analysis, particularly single-frequency BIA, it should be interpreted as an indirect, model-derived estimate that reflects intracellular water volume and general aspects of body composition, including muscle mass and overall hydration, rather than a specific physiological measure [6, 7]. Because older adults differ in body size and composition, hydration estimates relative to body mass provide additional context. The ratio of total body water to body weight (TBW/BW percent) should not be interpreted as equivalent to ICW, since it includes both intracellular and extracellular compartments, which can vary according to individual physiological conditions. TBW/BW percent functions as a size-adjusted indicator of overall hydration status and helps situate intracellular estimates within a broader view of fluid balance. Taken together, ICW and TBW/BW percent provide distinct but complementary information on hydration patterns and body composition [7].

In this context, bioelectrical impedance analysis has gained prominence as a non-invasive, accessible, and cost-effective tool for estimating hydration-related parameters in older populations [8–10]. In addition to providing estimates of fat and fat-free mass, BIA enables indirect quantification of fluid compartments such as ICW and TBW/BW%. However, its feasibility may be limited in individuals with severe edema, implanted electronic devices, or mobility impairments that hinder proper positioning. Despite such limitations, BIA offers a practical means for detecting hydration-related alterations in primary care settings, where simple and scalable screening instruments are essential for geriatric risk assessment [8, 9, 11].

Recent evidence [10, 12] indicates that BIA-derived estimates of ICW and relative hydration (e.g., TBW/BW%) are associated with muscle strength, physical performance, and frailty-related outcomes in older adults [6, 7, 13]. However, few studies have examined these variables jointly or explored their independent contributions across multiple functional outcomes using robust multivariate models that also address potential collinearity between hydration parameters.

To better understand the complex interrelationships among body water compartments, muscle characteristics, and physical function, path analysis provides an appropriate analytical framework. This approach enables simultaneous evaluation of several associative pathways, offering a more nuanced understanding of how hydration-related estimates may relate to muscle strength, muscle mass, and functional capacity in older adults [14, 15].

In this study, we focused on ICW as an indirect hydration-related estimate derived from BIA that is generally interpreted as reflecting the metabolically active component of body mass and is often used as a proxy for muscle cell hydration. Although extracellular water and the ICW-to-ECW ratio also offer relevant information, ICW was prioritized to maintain conceptual clarity and reduce multicollinearity, allowing a clearer examination of its unique statistical associations with functional outcomes. By examining ICW and TBW/BW% in relation to multiple indicators of physical function—handgrip strength, calf circumference as a proxy for muscle mass, and the Short Physical Performance Battery (SPPB)—this study contributes to existing evidence by evaluating their concurrent and independent relationships through path analysis. This analytical approach enables simultaneous assessment of direct and indirect associations while accounting for shared variance between hydration-related measures.

Considering these aspects, the present study aimed to investigate the associations between intracellular water and relative hydration status, assessed through the TBW-to-body-weight ratio, and indicators of muscle strength (handgrip strength), muscle mass proxy (calf circumference), and physical performance (SPPB) in community-dwelling older adults. Using path analysis, we examined how these hydration-related estimates relate to functional outcomes. The findings may support the refinement of screening and monitoring strategies in geriatric care, with the potential to enhance early identification of functional vulnerability and promote healthy aging.

## Material and methods

### Study design and setting

This cross-sectional observational study was conducted in the primary health care units in Parnamirim, Rio Grande do Norte, Brazil. The study followed the main recommendations of the Strengthening the Reporting of Observational Studies in Epidemiology (STROBE) [16] guidelines and was approved by the Research Ethics Committee of the Onofre Lopes University Hospital (HUOL/UFRN), under approval number 2,996,329.

### Participants

The sample consisted of older adults enrolled in the "Pro – Eva" study (Promoção do Envelhecimento e Vida Ativa) [17]. Data collection took place at the primary health care units of Parnamirim, RN, Brazil. Participants were recruited through coordination with the Municipal Health Department, which facilitated contact with the managers of each unit. Eligible older adults were invited to participate during routine visits to the health units.

Inclusion criteria were: community-dwelling adults aged ≥ 60 years, of both sexes, registered at local primary care units, and without cognitive impairment, defined as ≤ 3 errors in the orientation domain of the Leganés Cognitive Test [18].

Exclusion criteria included decompensated neuromuscular, cardiovascular, pulmonary, renal, or hepatic diseases, and conditions that could affect hydration or the feasibility of BIA, such as severe edema, implanted electronic devices, or inability to assume the supine position.

### Procedures

#### Sociodemographic variables

All assessments were performed by trained physiotherapists.

Interviewers underwent standardized training to ensure procedural reliability and adherence to study protocols. Sociodemographic and clinical data were collected through structured interviews and verified in medical records when available. Clinical variables included self-reported diagnoses of hypertension and diabetes, and polypharmacy, defined as the use of five or more medications, a widely adopted cutoff in geriatric research [17].

#### Anthropometric variables

Height (m) was measured using a fixed wall-mounted stadiometer, and weight (kg) with a calibrated digital scale (Glass 10, G-Tech®), following standard techniques (WHO, 2008). Participants stood barefoot, with heels together and body aligned with the device.

Body mass index (BMI) was calculated as weight (kg)/height (m)^2^. Nutritional status was classified as underweight (< 18.5 kg/m^2^), eutrophic (18.5–24.9 kg/m^2^), overweight (25–29.9 kg/m^2^), or obese (≥ 30 kg/m^2^), based on WHO and Nuttall [19].

Calf circumference (CC) was assessed using a non-elastic tape, at the maximum circumference of the left calf, measured in the standing position with both feet 20 cm apart [20].

#### Body composition

Body composition parameters, including intracellular water (ICW), total body water (TBW), and basal metabolic rate, were assessed using a single-frequency bioelectrical impedance analyzer (Biodynamics, model 450; 50 kHz). This device estimates tissue properties from impedance and reactance using proprietary prediction algorithms embedded in the equipment [21]. Participants were instructed to avoid high-calorie meals, alcohol, and caffeine for at least 4 h, refrain from vigorous exercise for 12 h, and maintain usual hydration by drinking water before the assessment. Measurements were conducted in the supine position using a tetrapolar configuration, with electrodes placed on the right hand and right foot. A conductive gel was applied to each site to minimize skin–electrode resistance and ensure stable signal acquisition.

The device provided model-derived estimates of intracellular water (ICW, kg) and total body water (TBW, kg), from which the total body water-to-weight ratio (TBW/BW, %) was calculated (TBW ÷ body weight × 100). In this study, ICW is interpreted as an indirect BIA-derived estimate related to intracellular hydration and the fluid component of fat-free/muscle mass, while TBW/BW% is considered a size-adjusted indicator of overall hydration status. These parameters offer complementary information about hydration patterns that may be associated with muscle and functional performance in older adults [9]. All body water parameters were automatically generated by the device in kilograms, without any manual conversion by the authors.

#### Handgrip strength

To measure handgrip strength, a Saehan® hydraulic dynamometer was used, which records muscle strength in the unit of kilograms/force (Kgf), measured based on the recommendations of the American Society of Hand Therapists (SATM) [22]. Thus, the participants were asked to squeeze the equipment with maximum isometric force, without any type of help and/or body movement for 5 s. For the present study, muscle strength was considered the average of the three attempts made [22].

#### Functional performance

Functional performance was assessed using the Short Physical Performance Battery (SPPB), a validated instrument for evaluating lower limb function in older adults [23]. The SPPB consists of three components: lower limb muscle strength (measured by the time to sit and get up from a chair), walking speed (time to cover a given distance), and balance (in three positions: feet together, semi-tandem, and tandem) [23].

The total SPPB score ranges from 0 to 12, with higher scores indicating better physical function [24].

#### Data analysis

Descriptive statistics were conducted to characterize the sample and clinical variables. Continuous variables are presented as means and standard deviations or medians (IQR) when non-normally distributed, and categorical variables as absolute and relative frequencies. Normality of distribution was assessed using the Shapiro–Wilk test.

Correlation analysis was performed to examine associations between physical function, anthropometric measures, and body composition parameters, considering that some variables did not meet the assumptions for parametric tests. Path analysis (structural equation modeling) was used to examine direct and indirect associations between clinical and body composition variables and the outcomes.

Each model included:

- Model 1: Predictors of HGS (sex, ICW, TBW/BW%, HBP, polypharmacy)
- Model 2: Predictors of CC (ICW, BMI, diabetes)
- Model 3: Predictors of SPPB (ICW, TBW/BW%, education, HBP, polypharmacy)

Standardized path coefficients (β) were estimated with robust maximum likelihood (FIML) and 95% confidence intervals. Goodness-of-fit was evaluated using χ^2^/df, SRMR, RMSEA, CFI, and TLI. Significance was set at *p* < 0.05. All analyses were conducted using JASP version 0.18.3.0.

## Results

From an initial pool of 1.336 eligible individuals, 1.009 completed the full protocol and were included in the final analysis. The sample consisted predominantly of older adults with a mean age of 70.3 ± 7.0 years, of whom 61.3% were women. Mean values of calf circumference, handgrip strength, intracellular water (ICW), and total body water-to-weight ratio (TBW/BW%) were examined by sex. No significant differences were observed between men and women for these parameters. The mean body mass index was 28 ± 4.9 kg/m^2^, and the mean calf circumference was 33.4 ± 3.7 cm. In addition, the mean muscle strength was 24.9 ± 8.2 kgf, the physical performance assessed by the SPPB was 9.7 ± 2.3 points. The other characteristics of the sample can be seen in Table 1.

**Table 1:** Demographic characteristics, body composition, and functional outcomes of 1.009 community-dwelling older adults from Parnamirim, RN, Brazil

Variables		n*	%	Mean	Standard Deviation
Gender	Male	390	38.7	-	-
	Female	619	61.3	-	-
Schooling	0–3 years	495	49.4	-	-
	Above 3 years	507	50.6	-	-
Polypharmacy	No	775	83.8	-	-
	Yes	150	16.2	-	-
HBP	No	396	41.5	-	-
	Yes	558	58.5	-	-
Diabetes melitus	No	689	71.0	-	-
	Yes	281	29.0	-	-
Age (years)		-	-	70.3	7.0
Body Mass Index (kg/m^2^)		-	-	28.0	4.9
Nutritional status (BMI)					
Underweight (< 18.5)		12	1.2	-	-
Eutrophic (18.5–24.9)		210	20.8	-	-
Overweight (25–29.9)		430	42.6	-	-
Obese (≥ 30)		357	35.4	-	-
ICW (kg)		-	-	17.0	3.9
TBW/BW (%)		-	-	48.3	5.1
Calf circumference (cm)		-	-	33.4	3.7
Score SPPB		-	-	9.7	2.3
Handgrip strength (kgf)		-	-	24.9	8.2

*BMI* Body mass index, *BIA* Bioelectrical impedance, *TBW* Total body water, *ICW* Intracellular water, *HBP* High blood pressure, *SPPB* Short Physical Performance Battery

n*: Only valid cases

Table 2 presents the correlation matrix among physical function, anthropometric measures, and body composition parameters. Overall, significant positive correlations were observed between hydration-related variables (ICW and TBW/BW) and physical performance measures, particularly handgrip strength and SPPB. Conversely, age showed weak or non-significant associations with most outcomes.

**Table 2:** Correlation analysis between physical function, anthropometric measures, and body composition in older adults, Parnamirim, Rio Grande do Norte (RN), Brazil

Variables		handgrip strength	SPPB	Calf circumference
Age	Spearman’s rho (ρ)	−0.04	−0.02	−0.06
	p	0.21	0.47	0.04
BMI	Spearman’s rho (ρ)	0.35	0.22	0.63
	p	< 0.001	< 0.001	< 0.001
ICW	Spearman’s rho (ρ)	0.64	0.29	0.43
	p	< 0.01	< 0.01	< 0.01
TBW/BW (%)	Spearman’s rho (ρ)	0.38	0.25	−0,21
	p	< 0.001	< 0.001	0.001

*BMI* Body mass index, *BIA* Bioelectrical impedance, *TBW* Total body water, *ICW* Intracellular water, *SPPB* Short Physical Performance Battery

**Fig. 1:**
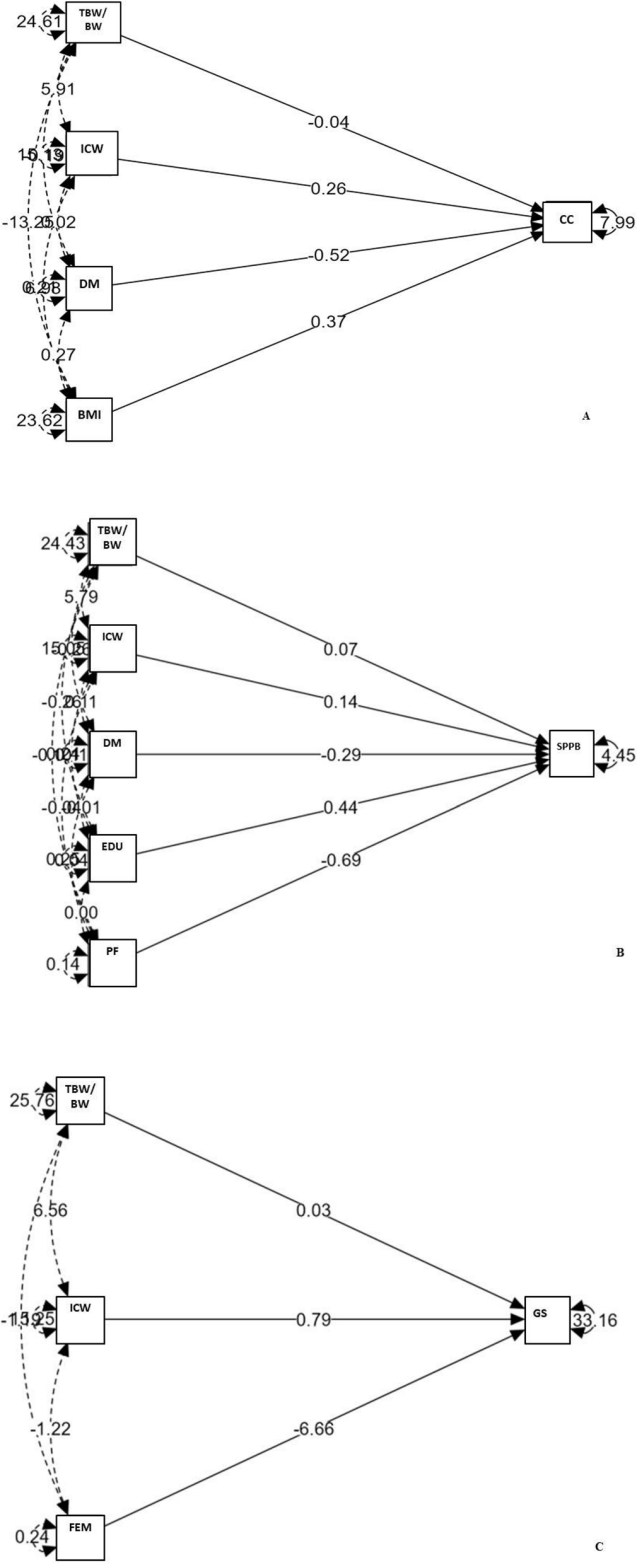
Graphical representation of the associations of clinical variables and body water on the outcomes. **A** Calf Circumference; **B** SPPB (Short Physical Performance Battery); **C** Handgrip Strength. Note: BMI = Body mass index; BIA = Bioelectrical impedance; TBW = Total body water; ICW = Intracellular water; HBP = High blood pressure; SPPB = Short Physical Performance Battery; DM = Diabetes Mellitus; FEM = Female; GS = grip strength; EDU = Education; PF = Polypharmacy

Structural equation modeling (SEM) was used to examine the statistical associations between clinical and body composition variables and three functional outcomes: handgrip strength, calf circumference, and physical performance (SPPB). Initially, predictor variables with a statistical significance of p < 0.10 in the bivariate analysis were included, along with ICW and TBW/BW (%). Subsequent adjustments were made, and the final models are presented. The standardized beta (β) values for the associations are shown in the figures corresponding to each model (Fig. 1 A, B, and C) and summarized in Table 3, along with their respective 95% confidence intervals.

**Table 3:** Results of the structural equation modeling (SEM) analysis

Predictors	β (95% IC)	*p*-value	R^2^
Final model of the calf circumference			
BMI	0.37 (0.31; 0.43)	< 0.001	42.1%
Diabetes mellitus (Yes)	−0.52 (−0.87; −0.18)	0.004	
ICW	0.26 (0.19; 0.32)	< 0.001	
TBW/BW (%)	−0.04 (−0.10; 0.01)	0.13	
Final model of the SPPB			
Polypharmacy (Yes)	−0.69 (−1.07; −0.30)	0.005	14.2%
HBP	−0.29 (−0,58; −0.05)	0.001	
ICW	0.14 (0,09; 0.18)	< 0.001	
TBW/BW (%)	0.07 (0.04; 0.10)	< 0.001	
Education (≥ 4 years)	0.44 (0.15; 0.71)	0.002	
Final model of handgrip strength			
Gender (Female)	−6.66 (−7.85; −5.88)	< 0.001	51.0%
ICW	0.79 (0.67; 0.92)	< 0.001	
TBW/BW (%)	0.03 (−0.04; 0.11)	0.41	

*BMI* Body mass index, *BIA* Bioelectrical impedance, *TBW* Total body water; *ICW* Intracellular water, *HBP* High blood pressure, *SPPB* Short Physical Performance Battery

n*: Only valid cases

In the final models, ICW remained a consistent and significant associated factor across all three outcomes. For calf circumference, BMI also contributed positively, while the TBW/BW (%) showed no significant effect. In the SPPB model, polypharmacy and HBP were negatively associated with performance, whereas schooling and relative hydration were positively related. For handgrip strength, sex and ICW showed the strongest associations.

## Discussion

This study identified associations between intracellular water (ICW) and relative hydration status, assessed through the ratio of total body water to body weight (TBW/BW percent) using bioimpedance analysis, and selected functional and anthropometric measures in community-dwelling older adults. ICW, obtained indirectly from single-frequency BIA, showed consistent associations with handgrip strength, calf circumference, and physical performance. These results represent statistical relationships only and do not validate ICW as a physiological marker of cellular integrity, muscle mass, or metabolic activity [25, 26].

The mean values observed for ICW (~ 17 kg) and TBW/BW% (~ 48%) align with previously reported ranges in older adult populations. These findings are compatible with age-related shifts in body composition and hydration patterns described in prior literature, particularly among women and individuals with reduced fat-free mass [27–29].

The correlation analysis demonstrated that hydration-related parameters were moderately associated with functional outcomes, echoing patterns reported in previous studies [30–33]. ICW showed the strongest associations with muscle strength and calf circumference; however, these relationships should be interpreted as reflecting general links between hydration-related body-composition parameters and functional status, rather than indicating any direct measure of muscle tissue characteristics or underlying cellular properties [34–36]. TBW/BW% demonstrated weaker but positive associations with physical performance, suggesting that different hydration indicators may relate to functional status through broad and nonspecific pathways that cannot be interpreted as physiological mechanisms.

Across the regression models, ICW was associated with all three functional outcomes, although the proportion of explained variance was modest—especially for SPPB (R^2^ = 14.2%). For handgrip strength and calf circumference, ICW and TBW/BW percent were statistically associated with these outcomes, although other variables such as sex, diabetes mellitus, and BMI showed stronger standardized associations [37–39]. These results reinforce that hydration-related parameters contribute to, but do not dominate, the complex set of factors influencing functional performance in older adults. TBW/BW% was associated only with SPPB, suggesting it may reflect broader aspects of body fluid distribution rather than specific muscular characteristics [40].

In the SPPB model, both ICW and TBW/BW% were positively associated with performance [40]. Polypharmacy and hypertension were negatively associated, consistent with their known relationships to poorer physical function in aging populations. Higher education levels were associated with better performance, aligning with established evidence linking educational attainment to healthier lifestyle patterns and more favorable functional trajectories [41].

The handgrip strength model explained over half of the variance, with sex having the largest effect, as expected [42–45]. ICW showed a positive association, reflecting its link with overall body composition rather than a direct physiological measure of muscle function. In the calf circumference model, ICW and BMI remained significant, indicating that both hydration-related and anthropometric factors shape this measure [44–47]. Diabetes was negatively associated with calf circumference, consistent with known associations between long-term metabolic dysregulation and reduced muscularity [44–47].

Taken together, these results add evidence that hydration-related parameters obtained through BIA, particularly ICW, are statistically associated with functional and anthropometric measures in older adults. However, they should be interpreted as indirect indicators of overall body composition and hydration, rather than as direct markers of cellular integrity or muscle function. These findings suggest that including hydration-related variables in geriatric assessments may provide complementary information, while avoiding causal or mechanistic interpretations.

### Strengths and limitations

Strengths of this study include its large community-based sample and the use of path analysis, which enabled the examination of multivariate associations while reducing collinearity. ICW in this context primarily reflects overall body size and muscle mass rather than isolated cellular hydration. Since single-frequency BIA cannot separate hydration from muscle mass, ICW should be considered a general body-composition indicator rather than a specific measure of muscle function. Together, these methodological features contribute to internal consistency and support the broader applicability of the findings [48–53]. Nonetheless, the cross-sectional design precludes temporal or causal interpretations, and the use of convenience sampling limits generalizability. The use of a single-frequency BIA device, while practical, reduces the precision of distinguishing ICW from ECW compartments [54–57]. Important behavioral variables such as physical activity, dietary intake, alcohol consumption, smoking, and general health status were not included and may confound the observed associations with hydration and muscle-related measures. Handgrip strength and calf circumference were analyzed separately to reduce multicollinearity, but their physiological interdependence should be considered when interpreting the results. Future longitudinal and stratified studies by sex and age are needed to clarify the temporal patterns and strengthen the interpretation of these associations.

## Conclusion

In conclusion, intracellular water and the total body water-to-weight ratio showed independent statistical associations with handgrip strength, calf circumference, and physical performance in community-dwelling older adults. ICW showed the strongest and most consistent associations, but it should be considered an indirect, BIA-derived estimate of general body composition and hydration. Although the explanatory power of the models was moderate, hydration-related measures may offer complementary information when evaluating functional status in older adults. Further longitudinal research is needed to clarify the temporal nature and clinical relevance of these associations.

## Data Availability

No datasets were generated or analysed during the current study.

## References

[1] Izquierdo M, Merchant RA, Morley JE, Anker SD, Aprahamian I, Arai H, et al. International exercise recommendations in older adults (ICFSR): expert consensus guidelines. J Nutr Health Aging. 2021;25(7):824–53. 10.1007/s12603-021-1665-8. 34409961 10.1007/s12603-021-1665-8 PMC12369211

[2] Volkert D, Beck AM, Cederholm T, Cruz-Jentoft A, Hooper L, Kiesswetter E, et al. ESPEN practical guideline: clinical nutrition and hydration in geriatrics. Clin Nutr. 2022;41(4):958–89. 10.1016/j.clnu.2022.01.024. 35306388 10.1016/j.clnu.2022.01.024

[3] Rodrigues F, Domingos C, Monteiro D, Morouço P. A review on aging, sarcopenia, falls, and resistance training in community-dwelling older adults. Int J Environ Res Public Health. 2022;19(2):874. 10.3390/ijerph19020874. 35055695 10.3390/ijerph19020874 PMC8775372

[4] Newman AB, Visser M, Kritchevsky SB, Simonsick E, Cawthon PM, Harris TB. The health, aging, and body composition (Health ABC) study—ground-breaking science for 25 years and counting. J Gerontol A Biol Sci Med Sci. 2023;78(11):2024–34. 10.1093/gerona/glad167. 37431156 10.1093/gerona/glad167 PMC10613019

[5] Potok OA, Ix JH, Shlipak MG, Bansal N, Katz R, Kritchevsky SB, et al. Cystatin C-and creatinine-based glomerular filtration rate estimation differences and muscle quantity and functional status in older adults: the health, aging, and body composition study. Kidney Med. 2022;4(3):100416. 10.1016/j.xkme.2022.100416. 35386603 10.1016/j.xkme.2022.100416 PMC8978136

[6] Serra-Prat M, Lorenzo I, Palomera E, Yébenes JC, Campins L, Cabré M. Intracellular water content in lean mass is associated with muscle strength, functional capacity, and frailty in community-dwelling elderly individuals. A cross-sectional study. Nutrients. 2019;11(3):661. 10.3390/nu11030661. 30893821 10.3390/nu11030661 PMC6471552

[7] Serra-Prat M, Lorenzo I, Papiol M, Palomera E, Bartolomé M, Pleguezuelos E, et al. Intracellular water content in lean mass as an indicator of muscle quality in an older obese population. J Clin Med. 2020;9(5):1580. 10.3390/jcm9051580. 32455974 10.3390/jcm9051580 PMC7290582

[8] Smith S, Madden AM. Body composition and functional assessment of nutritional status in adults: a narrative review of imaging, impedance, strength and functional techniques. J Hum Nutr Diet. 2016;29(6):714–32. 10.1111/jhn.12372. 27137882 10.1111/jhn.12372

[9] Roubenoff R, Baumgartner RN, Harris TB, Dallal GE, Hannan MT, Economos CD, et al. Application of bioelectrical impedance analysis to elderly populations. J Gerontol A Biol Sci Med Sci. 1997;52(3):M129–36. 10.1093/gerona/52a.3.m129. 9158553 10.1093/gerona/52a.3.m129

[10] Sales WB, Fernandes SGG, Gonçalves RSDSA, de Andrade LEL, Ramalho CST, de Souza GF, et al. Use of electrical bioimpedance in the assessment of sarcopenia in the older adults: A scoping review. J Bodyw Mov Ther. 2024. 10.1016/j.jbmt.2024.02.015. 38876654 10.1016/j.jbmt.2024.02.015

[11] Barbosa-Silva AJ, Barros J, Wang SB, Heymsfield RN, Pierson Jr. Bioelectrical impedance analysis: population reference values for phase angle by age and sex. Am J Clin Nutr. 2005;82(1):49–52. 10.1093/ajcn.82.1.49. 16002799 10.1093/ajcn.82.1.49

[12] Okura T, Asano Y, Tsuji T. Segmental extracellular-to-intracellular water resistance ratio and physical function in older adults. Exp Gerontol. 2023;181:112278. 10.1016/j.exger.2023.112278. 37597709 10.1016/j.exger.2023.112278

[13] Carey DG, Pliego GJ, Raymond RL. Body composition and metabolic changes following bariatric surgery: effects on fat mass, lean mass and basal metabolic rate: six months to one-year follow-up. Obes Surg. 2006;16(12):1602–8. 10.1381/096089206779319347. 17217636 10.1381/096089206779319347

[14] Schumacker RE, Lomax RG. A Beginner’s Guide to Structural Equation Modeling. 4th ed. New York: Routledge; 2016.

[15] Hyvärinen M, Kankaanpää A, Rantalainen T, et al. Body composition and functional capacity as determinants of physical activity in middle-aged and older adults: a cross-sectional analysis. Eur Rev Aging Phys Act. 2025;22:6. 10.1186/s11556-025-00372-z. 40312657 10.1186/s11556-025-00372-z PMC12044818

[16] Von Elm E, Altman DG, Egger M, Pocock SJ, Gøtzsche PC, Vandenbroucke JP, et al. The Strengthening the Reporting of Observational Studies in Epidemiology (STROBE) Statement: guidelines for reporting observational studies. Int J Surg. 2014;12(12):1495–9. 10.1016/j.jclinepi.2007.11.008. 17947786 10.1136/bmj.39335.541782.AD PMC2034723

[17] Gonçalves RS, de Andrade LEL, Fernandes SG, de Albuquerque IS, Guerra RO, Maciel ÁC. Relato de experiência e resultados preliminares do estudo pro-eva: uma proposta para o manejo da caderneta de saúde da pessoa idosa. Estudos Interdisciplinares sobre o Envelhecimento. 2022;27(1). 10.22456/2316-2171.105228.

[18] Caldas VVDA, Zunzunegui MV, Freire ADNF, Guerra RO. Tradução, adaptação cultural e avaliação psicométrica da prova cognitiva de Leganés em uma população idosa brasileira com baixo nível educacional. Arq Neuropsiquiatr. 2012;70:22–7. 10.1590/S0004-282X2012000100006. 22218469 10.1590/s0004-282x2012000100006

[19] Nuttall FQ. Body mass index: obesity, BMI, and health: a critical review. Nutr Today. 2015;50(3):117–28. 10.1097/NT.0000000000000092. 27340299 10.1097/NT.0000000000000092 PMC4890841

[20] Landi F, Onder G, Russo A, Liperoti R, Tosato M, Martone AM, et al. Calf circumference, frailty and physical performance among older adults living in the community. Clin Nutr. 2014;33(3):539–44. 10.1016/j.clnu.2013.07.013. 23948128 10.1016/j.clnu.2013.07.013

[21] Kiefer LS, Fabian J, Rospleszcz S, Lorbeer R, Machann J, Kraus MS, et al. Population-based cohort imaging: skeletal muscle mass by magnetic resonance imaging in correlation to bioelectrical-impedance analysis. J Cachexia Sarcopenia Muscle. 2022;13(2):976–86. 10.1002/jcsm.12913. 35080141 10.1002/jcsm.12913 PMC8977960

[22] Reis MM, Arantes PMM. Medida da força de preensão manual-validade e confiabilidade do dinamômetro saehan. Fisioter Pesqui. 2011;18:176–81. 10.1590/S1809-29502011000200013.

[23] Treacy D, Hassett L. The short physical performance battery. J Physiother. 2018;64(1):61. 10.1016/j.jphys.2017.04.002. 28645532 10.1016/j.jphys.2017.04.002

[24] Ramírez-Vélez R, De Asteasu MLS, Morley JE, Cano-Gutierrez CA, Izquierdo M. Performance of the short physical performance battery in identifying the frailty phenotype and predicting geriatric syndromes in community-dwelling elderly. J Nutr Health Aging. 2021;25(2):209–17. 10.1007/s12603-020-1484-3. 33491036 10.1007/s12603-020-1484-3 PMC12876728

[25] Akamatsu Y, Kusakabe T, Arai H, Yamamoto Y, Nakao K, Ikeue K, et al. Phase angle from bioelectrical impedance analysis is a useful indicator of muscle quality. J Cachexia Sarcopenia Muscle. 2022;13(1):180–9. 10.1002/jcsm.12860. 34845859 10.1002/jcsm.12860 PMC8818694

[26] Asano Y, Tsuji T, Okura T. Segmental extracellular-to-intracellular water resistance ratio and physical function in older adults. Exp Gerontol. 2023;181:112278. 10.1016/j.exger.2023.112278. 37597709 10.1016/j.exger.2023.112278

[27] Taniguchi M, Yamada Y, Fukumoto Y, Sawano S, Minami S, Ikezoe T, et al. Increase in echo intensity and extracellular-to-intracellular water ratio is independently associated with muscle weakness in elderly women. Eur J Appl Physiol. 2017;117:2001–7. 10.1007/s00421-017-3686-x. 28755131 10.1007/s00421-017-3686-x

[28] Bahat G. Measuring calf circumference: a practical tool to predict skeletal muscle mass via adjustment with BMI. Am J Clin Nutr. 2021;113(6):1398–9 S0002-9165(23)07238-6. 33876186 10.1093/ajcn/nqab107

[29] Bennouar S, Bachir Cherif A, Raaf N, Hani HM, Kessira A, Abdi S. Raw bioelectrical impedance parameters and vector analysis in the screening of low muscle mass and low muscle mass associated with obesity in adult healthy subjects. Intern Emerg Med. 2025. 10.1007/s11739-025-03857-y. 39812907 10.1007/s11739-025-03857-y

[30] Min JY, Min KB. Comparisons of two bioelectrical impedance devices and manual versus sensor-based short physical performance batteries for assessment of muscle mass and physical performance. Sensors. 2023;23(13):6026. 10.3390/s23136026. 37447873 10.3390/s23136026 PMC10346212

[31] Martins PC, Junior CASA, Silva AM, Silva DAS. Phase angle and body composition: a scoping review. Clin Nutr ESPEN. 2023;56:237–50. 10.1016/j.clnesp.2023.05.015. 37344079 10.1016/j.clnesp.2023.05.015

[32] Canda AS. Puntos de corte de diferentes parámetros antropométricos para el diagnóstico de sarcopenia. Nutr Hosp. 2015;32(2):765–70. 10.3305/nh.2015.32.2.9193. 26268109 10.3305/nh.2015.32.2.9193

[33] Chang SH, Beason TS, Hunleth JM, Colditz GA. A systematic review of body fat distribution and mortality in older people. Maturitas. 2012;72(3):175–91. 10.1016/j.maturitas.2012.04.004. 22595204 10.1016/j.maturitas.2012.04.004 PMC3367099

[34] Iwasaka C, Yamada Y, Nishida Y, Hara M, Yasukata J, Miyoshi N, et al. Association between the appendicular extracellular-to-intracellular water ratio and all-cause mortality: a 10-year longitudinal study. J Gerontol A Biol Sci Med Sci. 2024;79(2):glad211. 10.1093/gerona/glad211. 37726006 10.1093/gerona/glad211 PMC10918756

[35] Oh S-K, et al. Association between basal metabolic rate and handgrip strength in older Koreans. Int J Environ Res Public Health. 2019;16(22):4377. 10.3390/ijerph16224377. 31717481 10.3390/ijerph16224377 PMC6888346

[36] Park K-S, et al. The relationship between extracellular water-to-body water ratio and sarcopenia according to the newly revised Asian Working Group for Sarcopenia: 2019 consensus update. Aging Clin Exp Res. 2021;33:2471–7. 10.1007/s40520-020-01766-y. 33454925 10.1007/s40520-020-01766-y

[37] Chen D, Fritz MS. Comparing alternative corrections for bias in the bias-corrected bootstrap test of mediation. Eval Health Prof. 2021;44(4):416–27. 10.1177/01632787211024356. 34142575 10.1177/01632787211024356 PMC8988747

[38] Cruz-Jentoft AJ, Bahat G, Bauer J, Boirie Y, Bruyère O, Cederholm T, et al. Sarcopenia: revised European consensus on definition and diagnosis. Age Ageing. 2019;48(1):16–31. 10.1093/ageing/afy169. 30312372 10.1093/ageing/afy169 PMC6322506

[39] Da Silva BR, Orsso CE, Gonzalez MC, et al. Phase angle and cellular health: inflammation and oxidative damage. Rev Endocr Metab Disord. 2023;24:543–62. 10.1007/s11154-022-09775-0. 36474107 10.1007/s11154-022-09775-0 PMC9735064

[40] Einhell S, Albrecht M, Windschüttl S, Sedlmeier AM, Lüke F, Sparrer D, et al. Physical performance, sarcopenia and malnutrition—basic test set for everyday use in cancer therapy. Cancer Med. 2026;15(1):e71505. 10.1002/cam4.71505 PMC12766154 41486820

[41] Dodds RM, Syddall HE, Cooper R, Benzeval M, Deary IJ, Dennison EM, et al. Grip strength across the life course: normative data from twelve British studies. PLoS One. 2014;9(12):e113637. 25474696 10.1371/journal.pone.0113637 PMC4256164

[42] Morley JE. Sarcopenia: diagnosis and treatment. J Nutr Health Aging. 2027;11(3):208–12. 10.1007/BF02982705. 10.1007/BF02982705 PMC12892578 18615226

[43] Oliveira C, Araujo ML, Diniz PRB, et al. Association of socioeconomic and lifestyle factors with physical performance in older Brazilian adults. BMC Geriatr. 2021;21:453.34348660

[44] Oliveira TDC, Gomes Fernandes SG, Silva dos Santos Aguiar Gonçalves R, Sales WB, de Andrade LEL, Vieira ER, Maciel ÁCC. Calf Circumference Cutoff Point for Sarcopenia Screening in Community-Dwelling Older Brazilians. Physical & Occupational Therapy In Geriatrics. 2024;1–13. 10.1080/02703181.2024.2388552.

[45] Onder G, Liperoti R, Fialová D, et al. Polypharmacy in nursing home in Europe: results from the SHELTER study. J Gerontol Med Sci. 2014;69(5):585–91. 10.1093/gerona/glr233 22219520

[46] Palmer K, Villani ER, Vetrano DL, Cherubini A, Cruz-Jentoft AJ, Curtin D, European Geriatric Medicine Society Pharmacology special interest group. Association of polypharmacy and hyperpolypharmacy with frailty states: a systematic review and meta-analysis. Eur Geriatr Med. 2019;10:9–36. 32720270 10.1007/s41999-018-0124-5

[47] Park SW, Goodpaster BH, Strotmeyer ES, et al. Decreased muscle strength and quality in older adults with type 2 diabetes: the Health, Aging, and Body Composition Study. Diabetes. 2006;55(6):1813–8. 16731847 10.2337/db05-1183

[48] Toselli S, Campa F, Matias CN, de Alencar Silva BS, Dos Santos VR, Latessa PM, et al. Predictive equation for assessing appendicular lean soft tissue mass using bioelectric impedance analysis in older adults: effect of body fat distribution. Exp Gerontol. 2021;150:111393. 10.1016/j.exger.2021.111393. 33965554 10.1016/j.exger.2021.111393

[49] Serra-Prat M, Lorenzo I, Papiol M, Palomera E, Bartolomé M, Pleguezuelos E, et al. Intracellular water content in lean mass as an indicator of muscle quality in an older obese population. J Clin Med. 2020;9(5):1580. 10.3390/jcm9051580. 10.3390/jcm9051580 PMC7290582 32455974

[50] Serra-Prat M, Lorenzo I, Palomera E, Yébenes JC, Campins L, Cabré M. Intracellular water content in lean mass is associated with muscle strength, functional capacity, and frailty in community-dwelling elderly individuals. A cross-sectional study. Nutrients. 2019;11(3):661. 10.3390/nu11030661. 10.3390/nu11030661 PMC6471552 30893821

[51] Scafoglieri A, Clarys JP, Bauer JM, Verlaan S, Van Malderen L, Vantieghem S, et al. Predicting appendicular lean and fat mass with bioelectrical impedance analysis in older adults with physical function decline–the PROVIDE study. Clin Nutr. 2017;36(3):869–75. 10.1016/j.clnu.2016.04.026. 27178302 10.1016/j.clnu.2016.04.026

[52] Ramírez-Vélez R, De Asteasu MLS, Morley JE, Cano-Gutierrez CA, Izquierdo M. Performance of the short physical performance battery in identifying the frailty phenotype and predicting geriatric syndromes in community-dwelling elderly. Journal Nutr Health Aging. 2021;25(2):209–17. 10.1007/s12603-020-1484-3. 10.1007/s12603-020-1484-3 PMC12876728 33491036

[53] Pereira JP, de Sousa Rebouças A, Prado CM, Gonzalez MC, Cabral PC, da Silva Diniz A, Silva FM. Phase angle as a marker of muscle quality: A systematic review and meta-analysis. Clin Nutr. 2024. 10.1016/j.clnu.2024.11.008. 39549478 10.1016/j.clnu.2024.11.008

[54] Piodena-Aportadera MRB, Lau S, Chew J, Lim JP, Ismail NH, Ding YY, et al. Calf circumference measurement protocols for sarcopenia screening: differences in agreement, convergent validity and diagnostic performance. Ann Geriatr Med Res. 2022;26(3):215. 10.4235/agmr.22.0057. 36031936 10.4235/agmr.22.0057 PMC9535367

[55] Rosano C, Newman AB, Katz R, Hirsch CH, Kuller LH. Association between lower education and worse cognitive and physical functioning in older adults. J Am Geriatr Soc. 2007;55(11):1741–6.17979897

[56] Veronese N, Stubbs B, Volpato S, et al. High blood pressure and frailty in community-dwelling older adults: cross-sectional and longitudinal data from the osteoarthritis initiative. J Am Med Dir Assoc. 2017;18(7):623–8.

[57] Volpato S, Bianchi L, Lauretani F, et al. Role of muscle mass and muscle quality in the association between diabetes and gait speed. Diabetes Care. 2012;35(8):1672–9. 22596176 10.2337/dc11-2202 PMC3402248

